# Magnetic compression for anastomosis in treating an infant born with long-gap oesophageal atresia: A case report

**DOI:** 10.1097/MD.0000000000022472

**Published:** 2020-10-16

**Authors:** Shi-Qi Liu, Yi Lv, Ying Fang, Rui-Xue Luo, Jing-Ru Zhao, Ruo-Gu Luo, Yi-Mei Li, Jing Zhang, Peng-Fei Zhang, Jin-Zhen Guo, Qing-Hong Li, Ming-Xing Han

**Affiliations:** a Medical College, Xijing University; b Department of Hepatobiliary Surgery, The First Affiliated Hospital of Xi’an Jiaotong University; c Xi’an Children's Hospital; d Northwest Institute for Nonferrous Metal Research (NIN); e Department of Pediatric; f Department of Neonatal Intensive Care Unit, The Northwest Women's and Children's Hospital, Xi’an, Shanxi, China.

**Keywords:** esophageal atresia, long-gap esophageal atresia, magnamosis, magnetic compression anastomosis, tracheoesophageal fistula

## Abstract

**Rationale:**

Neonatal long-gap esophageal atresia (LGEA) with tracheoesophageal fistula (TEF) is an uncommon but serious congenital malformation of the esophagus in newborns, and it remains challenging for pediatric surgeons. Magnetic compress has been shown to be effective for the treatment of LGEA in children and adults. However, the implementation of this unique technique for neonatal LGEA has not been evaluated.

**Patient concerns:**

A female infant was born at 37 weeks of gestation. Prenatal ultrasound imaging revealed signs of esophageal atresia, including the absence of the gastric bubble and polyhydramnios.

**Diagnoses:**

A diagnosis of LGEA with TEF was confirmed at birth by contrast X-ray.

**Interventions:**

She was treated with magnetic compression anastomosis (MCA) following an esophago-esophagostomy. Two magnetic rings were customized, and the MCA was conducted during the same stage surgery of ligating the TEF. Under the magnetic force, the 2 magnet rings pulled along the gastric tube to achieve anastomosis. The postoperative permanent suction of these 2 pouches was instituted, and spontaneous growth was awaited. Magnet removal was performed at 36 days, and enteral nutrition was continued via a gastric tube for 4 weeks at post-operation.

**Outcomes:**

The upper gastrointestinal contrast confirmed the anastomotic patency perfectly after 3 months. The patient was followed up for 18 months, and exhibited durable esophageal patency without dysphagia.

**Lessons:**

These results suggest that MCA is feasible and effective for treating LGEA in infants.

## 1 Introduction

Long-gap esophageal atresia (LGEA) with distal tracheoesophageal fistula (TEF) in newborns represents a rare but serous clinical condition. Several conventional surgical techniques, such as gastric or intestinal interposition and active elongation of the pouches, have been developed to establish the continuity of the gut.^[1]^

The present study describes a case of LGEA with TEF type IIIa in an infant, which was successfully treated with 1 surgical procedure for esophago-esophagostomy, which achieved by magnetic compression anastomosis (MCA) with thoracotomy and endoscopy.

## 2 Case presentation

**Figure 1:**
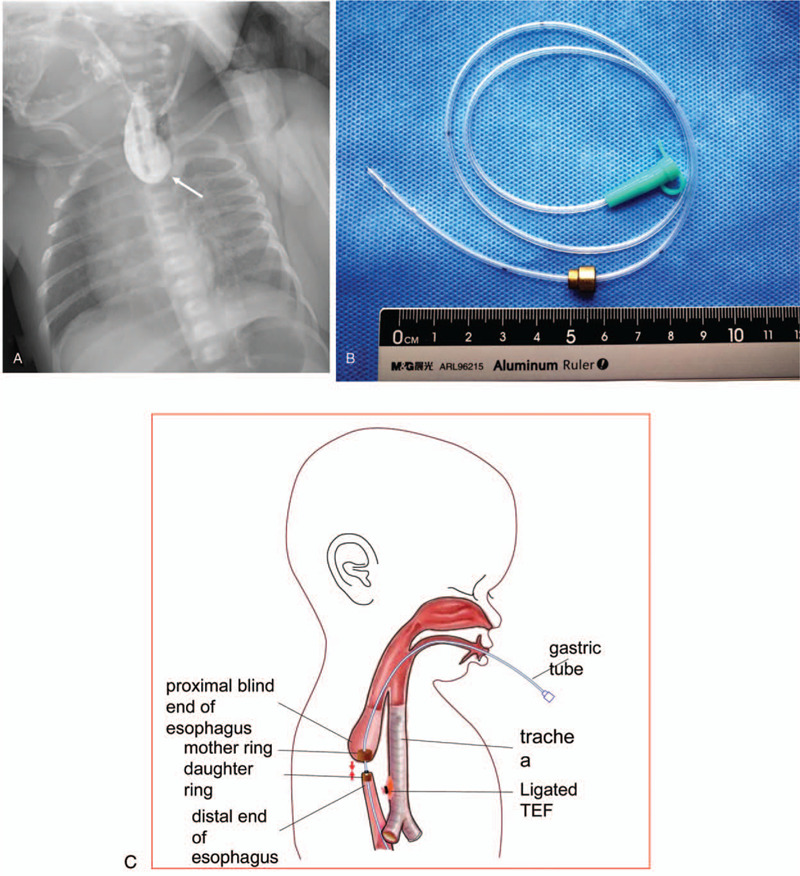
The preoperative X-ray image, design of the magnetic rings, and schematic presentation of the magnet rings to be placed for the patient is shown. (A) The anteroposterior X-ray image reveals that the proximal blind end of the esophagus lies at the level of the second thoracic vertebra. (B) The magnetic equipment designed and used for the present case is shown. The mother ring had an outer diameter of 7 mm and a thickness of 5 mm, while the daughter ring had an outer diameter of 5 mm and a thickness of 3 mm. (C) The schematic presentation of the axial magnet rings to be placed into the upper and distal ends of the esophagus, respectively. The mother ring was scheduled to be placed in the proximal end of the esophagus, while the daughter ring was planned to be positioned in the distal esophageal end. A 6F gastric tube was to be placed through the central hole of both rings and esophageal blind pouch until it reached the stomach cavity, and subsequently, the pouch suture distal esophageal. The contact of the 2 esophageal ends was anticipated to achieve esophageal anastomosis.

A female infant was born at 37 weeks of gestation and weighted 3200 g. The prenatal ultrasound imaging revealed signs of esophageal atresia (EA), including the absence of the gastric bubble and polyhydramnios. A diagnosis of LGEA with TEF was confirmed at birth by contrast X-ray. As shown in Figure 1A, the proximal end of the esophagus was located at the level of the second thoracic vertebra (IIIa type). The physical examination identified thumb polydactyly of both hands, and the echocardiography revealed patient foramen ovale and atrial septal defects.

The infants parents decided in agreement to attempt to approximate the 2 pouches by magnetic force, as previously reported.^[2]^ Written informed consent was obtained from the infants parents. This study was approved by the Ethics Committee of Northwest Women's and Children's Hospital (2017020).

The patient with LGEA and TEF (type IIIa) was scheduled for 1 surgical procedure of esophago-esophagostomy, in which magnetic compression was implemented to achieve anastomosis. Two magnetic rings with a strength of 2500 G force (Fig. 1B) were customized with a suction power between the esophageal upper and distal pouches (Fig. 1C). Briefly, the MCA was performed during the same stage surgery of ligating the TEF. One mother ring (7 × 5 mm) was placed in the proximal esophageal pouch under endoscopy, while the daughter ring (5 × 3 mm) was positioned in the distal esophageal lumen. Subsequently, a 6F gastric tube was inserted through the central hole of the mother ring and esophageal blind pouch under endoscopy. Then, this was allowed to go through the low ring in the distal esophageal until it reached the stomach cavity. Under the magnetic force, these 2 magnet rings pulled along the gastric tube to achieve esophageal anastomosis. The patient was fed via a gastric tube at post-operation.

The postoperative permanent suction of these 2 pouches was instituted, and the spontaneous growth of these 2 pouches was awaited. Enteral nutrition was continued via a gastric tube. Radiographic approximation was performed on day 1. A temporary increase in leucocyte count in peripheral blood to a maximum of 20.3 × 10^9^/L and C-reactive protein to 105 mg/l was observed, which normalized within 3 days.

**Figure 2:**
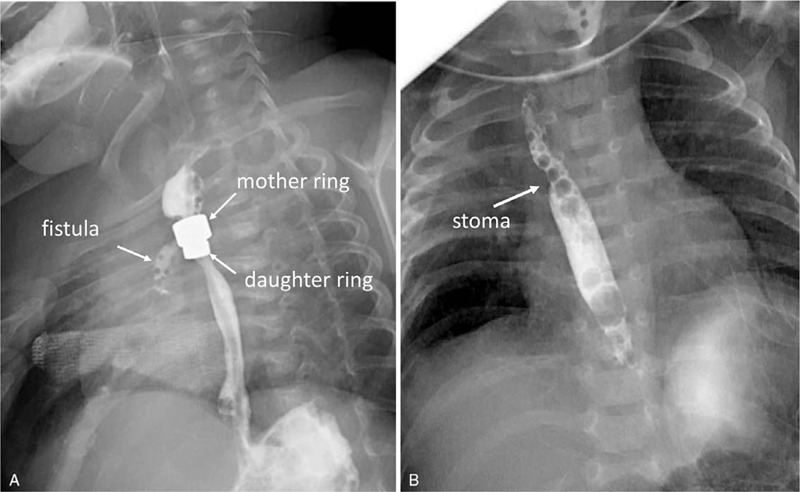
Serial postoperative radiographs of the X-ray images: (A) The esophagography confirmed the anastomotic fistula on day 7 after the surgery; (B) The esophagogram reveals that the magnet arrays were removed on day 36, and that the esophageal achieved restoration without leakage.

The magnet placement was successful, as determined when the patient was observed postoperatively in the neonatal intensive care unit (NICU). Anastomotic leakage occurred at 7 days, while healing was achieved on day 25 (Fig. 2A). Magnet removal was performed at 36 days post-operation, and the esophagram demonstrated the absence of perforations or other early complications (Fig. 2B). The upper gastrointestinal contrast confirmed the anastomotic patency perfectly after 3 months, and body-weight and height normally increased. The patient was followed up for 18 months, and exhibited durable esophageal patency without dysphagia.

## 3 Discussion

The treatment of LGEA remains challenging, which usually requires thoracotomy for the placement of traction sutures on each esophageal.^[3]^ MCA has been effectively used to achieve sutureless anastomoses in adults and children with LGEA. To the best of our knowledge, the present case study is the first to report a rare case for the feasibility and efficacy of MCA in achieving neonatal LEGA anastomosis.

The successful treatment of LGEA in the infant may merit attention. MCA was previously reported for treating benign biliary strictures,^[4]^ as magnetic connectors for coronary surgery,^[5]^ and for the functional undiversion of ileostomyin pediatric patients.^[6]^ At present, MCA for the treatment of EA patients is restricted to gross type A (without TEF) and anastomotic stenosis without thoracotomy.^[7]^ In the present case, the newborn patient was confirmed to have LGEA with an approximately 3-cm gap. Notably, anastomosis was achieved on day 36, exceeding the previously reported mean time of 4.2 to 6.0 days.^[8]^ Since the strength of the magnets affect the anastomosis time, the 2 rings should produce a magnetic compression power lower than 12,000 G.^[8]^ It is suggested that the time needed to have a solid anastomosis and allow for the easy removal of the magnets should range within 10 to 14 days, and this can be shortened if the field strength of the magnetic rings is increased.

For the magnamosis, the shape and size, when compared with the esophageal diameter and strength of magnets, may be important. For LGEA patients with a gap of more than 3 cm, the method could be a promising modality for the significant spontaneous growth and elongation of the pouches, which occurs within a few weeks postnatal.^[8]^ Further investigation and refinement of the method is required before this can be recommended in clinical practice.

Importantly, the attractive force between these 2 magnets exponentially increases as the distance of separation decreases. Due to this property of magnetism, there is a risk of tearing or perforation due to excessive force, since the esophageal gap decreases during treatment. This is the reason for the anastomatic fistula that occurred in the present case on day 7 after the operation.

Notably, the magnetic coupling occurred more rapidly than anticipated. Leaks appeared to be unavoidable in the patient due to the weaker wall of the esophagus in newborns, when compared to children. It is noteworthy that the patient developed a short-time anastomotic fistula, but no other severe short-term complications were observed.

## 4 Conclusions

In summary, the successful anastomosis with magnetic compression in the present case, who was born with LGEA, is worthy of attention. These findings suggest that MCA is a feasible and effective method for the treatment of LGEA in infants. MCA has been advocated for the treatment of such cases.

## Acknowledgments

We thank the Northwest Institute for Nonferrous Metal Research (NIN) for the Nv-Fe-P material processing and magnetic rings production. We are also grateful to the staff in the Medical Record Information Management Department for their support and assistance.

## Author contributions

Yi Lv and Jing-Ru Zhao guided the operation and revised the manuscript. Shi-Qi Liu conducted the operation and drafted this manuscript. Rui-Xue Luo and Ming-Xing Han assisted in the magnetic rings processing. Ying Fang contributed to the endoscopic procedures. Peng-Fei Zhang, Ruo-Gu Luo and Jing zhang provided assistance in the operation, and contributed to the perioperative management. Jin-Zhen Guo and Qing-Hong Li contributed to the NICU management. Yi-Mei Li was responsible for the patient's nursing. All authors have read and approved the final manuscript.
